# Repeated Automated Mobile Text Messaging Reminders for Follow-Up of Positive Fecal Occult Blood Tests: Randomized Controlled Trial

**DOI:** 10.2196/11114

**Published:** 2019-02-05

**Authors:** Revital Azulay, Liora Valinsky, Fabienne Hershkowitz, Racheli Magnezi

**Affiliations:** 1 Master of Health Administration Program, Department of Management Bar Ilan University Ramat Gan Israel; 2 Central Laboratory Meuhedet Health Care Lod Israel; 3 Quality Department Meuhedet Health Care Tel Aviv Israel

**Keywords:** adherence, cancer screening, colonoscopy, fetal occult blood test, patient-physician relationship, positive colorectal cancer screening, SMS, text reminder

## Abstract

**Background:**

Fecal occult blood tests (FOBTs) are recommended by the US Preventive Services Task Force as a screening method for colorectal cancer (CRC), but they are only effective if positive results are followed by colonoscopy. Surprisingly, a large proportion of patients with a positive result do not follow this recommendation.

**Objective:**

The objective of this study was to examine the effectiveness of text messaging (short message service, SMS) in increasing adherence to colonoscopy follow-up after a positive FOBT result.

**Methods:**

This randomized controlled trial was conducted with patients who had positive CRC screening results. Randomization was stratified by residential district and socioeconomic status (SES). Subjects in the control group (n=238) received routine care that included an alert to the physician regarding the positive FOBT result. The intervention group (n=232) received routine care and 3 text messaging SMS reminders to visit their primary care physician. Adherence to colonoscopy was measured 120 days from the positive result. All patient information, including test results and colonoscopy completion, were obtained from their electronic medical records. Physicians of study patients completed an attitude survey regarding FOBT as a screening test for CRC. Intervention and control group variables (dependent and independent) were compared using chi-square test. Logistic regression was used to calculate odds ratios (ORs) and 95% CIs for performing colonoscopy within 120 days for the intervention group compared with the control group while adjusting for potential confounders including age, gender, SES, district, ethnicity, and physicians’ attitude.

**Results:**

Overall, 163 of the 232 patients in the intervention group and 112 of the 238 patients in the control group underwent colonoscopy within 120 days of the positive FOBT results (70.3% vs 47.1%; OR 2.17, 95% CI 1.49-3.17; *P*<.001); this association remained significant after adjusting for potential confounders (*P*=.001).

**Conclusions:**

A text message (SMS) reminder is an effective, simple, and inexpensive method for improving adherence among patients with positive colorectal screening results. This type of intervention could also be evaluated for other types of screening tests.

**Trial Registration:**

ClinicalTrials.gov NCT03642652; https://clinicaltrials.gov/ct2/show/NCT03642652 (Archived by WebCite at http://www.webcitation.org/74TlICijl)

## Introduction

Colorectal cancer (CRC) is a major cause of morbidity and mortality throughout the world. It is the second most common malignant disease [1,2], with 90% survival when it is identified early and immediate surgical intervention is performed. In Israel, the screening policy for average-risk individuals aged 50-75 years is an annual fecal occult blood test (FOBT) [3]. A patient with positive FOBT result requires immediate follow-up with colonoscopy, and surgery should be performed when CRC is detected. A delay in follow-up markedly undermines the benefits of CRC screening, including incidence, mortality, life-years saved, and net costs of screening [4-7]. Recommendations regarding the time between a positive result and colonoscopy vary across countries, ranging between 30 and 180 days [8-13]. In Israel, the Ministry of Health guidelines define the standard period between a positive FOBT result and a follow-up colonoscopy as 90 days [3,14]. From the literature, we learned that 40%-60% of individuals who undergo FOBT screening do not continue with follow-up after a positive result [7,15,16]. In the Israeli population, follow-up rates after a positive FOBT result are 71%, and the proportion of patients who complete follow-up varies across health care organizations, with the proportion in Meuhedet being about 50%. The median time to follow-up, nationally, is 112 days, which is significantly longer than the recommended 90 days [17].

Barriers to follow-up after a positive FOBT result have been identified in the literature [18-23] and are divided into 4 general groups based on whether they are related to patients, physicians, providers, or information technology [24-26]. Intervention programs have been developed to target each of these groups [27]. Patient-targeted interventions include educational strategies, peer counselors or navigators, reminders, and coupons. The interventions were effective for short-term, but not long-term, follow-up. Several studies have described interventions aimed at increasing follow-up rates and have showed mixed results [13,20,28-41]. When the patient-physician relation is good and based on clear communication and trust, the patient is more likely to adhere to and fulfill the physician’s instructions [42,43].

Studies addressing patient interventions have used letters, emails, telephone calls, and nurse navigators. Mailed invitations were found to be as effective as telephone reminders and increased follow-up rates by approximately 30% in an Italian study [40]. In a Scottish study, a reminder phone call that included making an appointment for colonoscopy increased adherence by 4.7% [13]. In a British study aimed at minority populations, the study nurse called patients repeatedly, invited them to the clinic, and scheduled a colonoscopy appointment for each one; this intervention increased follow-up by 8.4% [39]. All these interventions were found to be effective, but some are extremely labor-intensive and time-consuming.

To date, few studies have examined the effectiveness of short message service (SMS) text messaging as an intervention tool within community medical settings. SMS text message reminders have been shown to be effective in improving preparation for colonoscopy in Korea [44]; they increased *Streptococcus pneumonia* immunization in a primary care setting in Lebanon [45] by 7.2% (less than phone calls, but more than emails). A literature review examining the effect of SMS text messages and emails to improve diabetes management showed that simple phone calls, letters, or SMS text message reminders can have a positive impact on clinical and behavioral outcomes [46]. A systematic review [47] indicated that SMS text messaging interventions improved patients’ medication adherence rate. In a review of the factors associated with nonadherence to oral antiplatelet therapy in acute coronary syndrome and interventions that modify these factors, only reminder-based interventions, including SMS text messages, had consistently beneficial impacts on adherence outcomes at 3 and 12 months [48]. In a randomized controlled trial designed to assess the effects of SMS text message reminders on adherence to a healthy diet, medication, and smoking cessation among adult patients with cardiovascular disease, researchers found that SMS text messaging was effective in improving adherence to a healthy diet and medication but not smoking cessation [49]. A different study reported increased success for smoking cessation among patients attending control visits as a result of scheduled clinic appointments following SMS text message reminders. The smoking cessation rate was 24% in patients who did not respond to SMS text message reminders at all and 28.6% (n=28) in patients answering any SMS text message at least once (*P*=.001) [50]. In contrast, in an Australian study, SMS text message reminders were used to improve hepatitis B vaccination among high-risk sexual health center attendees, and it was found that this intervention was not effective [51].

To the best of our knowledge, no study has used SMS text messaging technology to increase adherence to recommendations for CRC screening follow-up after a positive FOBT result. Therefore, we aimed to examine the effectiveness of sending SMS text messages to patients as an automated tool to increase adherence to colonoscopy follow-up and to examine the influence of physicians’ attitude toward FOBT as a screening test for CRC and patient adherence to follow-up.

## Methods

### Methodology

**Figure 1:**
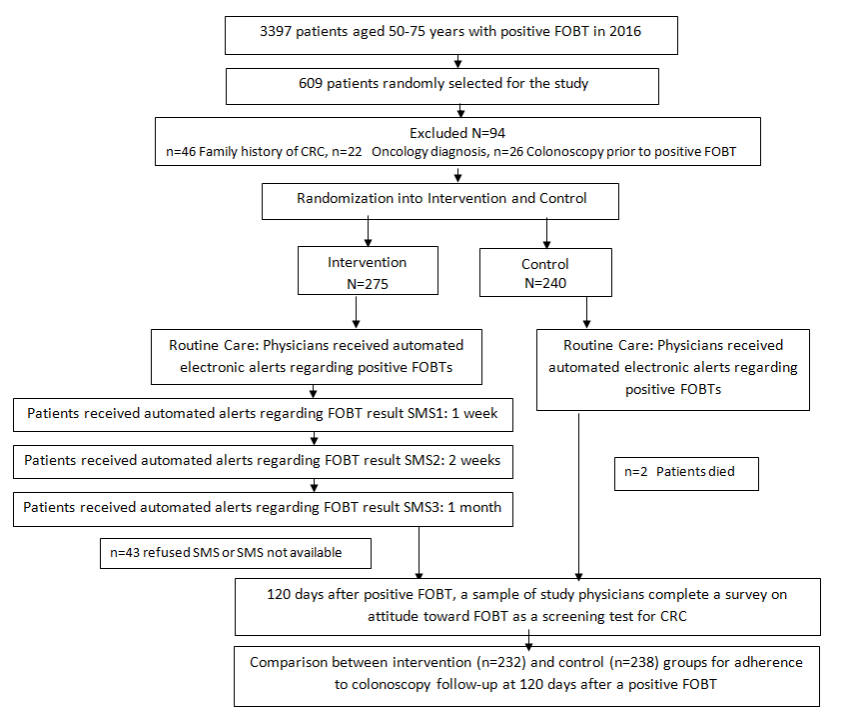
Study selection and design. FOBT: fecal occult blood test; CRC: colorectal cancer; SMS: short message service.

This study was conducted between January 2016 and March 2017 in Meuhedet, 1 of 4 health care organizations in Israel. It insures and provides care for 1.2 million members. The current rate of colorectal screening among 180,000 members aged 50-75 years is 60%, similar to the Israeli population. As per our data, in 2016, the rate of follow-up colonoscopy after a positive FOBT result in Meuhedet was 41%. The FOBT test used in Meuhedet is OC-SENSOR Immunochemical Kit (Eiken Chemical Co, Ltd, Tokyo, Japan). The study was approved by the Meuhedet Institutional Review Board on March 23, 2016 (trial reference number: 01-023-03-016). The study was exempted from informed consent, and only agreement to receive SMS text messages was required. Figure 1 describes the study flow (Multimedia Appendix 1 presents the CONSORT checklist [52]).

### Study Population

In 2016, 3397 patients aged 50-75 years had a positive FOBT result. Of them, 609 were randomly selected and, then, randomly allocated to the intervention or control group. The inclusion criteria included age 50-75 years and providing consent to receive SMS text messages from Meuhedet. By Israeli law, health messages via SMS text messages may only be sent to members who actively agree to receive them. The exclusion criteria were personal or family history of CRC, colonoscopy 10 years before the positive FOBT result, or diagnosis of any type of cancer during the study period.

### Physicians

All primary care physicians whose patients (control and intervention groups) had completed an FOBT during 2016 and were employed by Meuhedet were included in this study. Physicians no longer working at Meuhedet were excluded from the study. All primary care physicians were notified about the study.

### Study Variables

The independent variables were the intervention or control group and physician attitude to FOBT. In addition, we examined potential confounders such as gender, age, socioeconomic status (SES), ethnicity, and residential district. The dependent variable was adherence to colonoscopy within 120 days of a positive FOBT result. This information was obtained from the patients’ electronic medical records (EMRs). Positive FOBT results were obtained from the Meuhedet Central Laboratory. Patients’ demographics and clinical characteristics including gender, age, district, SES, and ethnicity were obtained from the EMRs. SMS text messages were sent to patients via InforUMobile software (Shamir Systems, 1974, Rishon LeZion) with a track whether the SMS text message was received or rejected.

### Study Protocol

Routine care for the control and intervention groups included an automated computer alert in a patient's EMR regarding the positive FOBT result, to his or her physician, with no indication of whether the patient had already visited the physician after the positive result. The automated alert was sent to physicians the moment the lab released the positive result. In addition, each patient in the intervention group received an automated SMS text message up to a week after the positive FOBT result. The text read: “Hello. There is a lab test result ready for you. Contact your physician for an explanation of the findings.” Furthermore, 2 additional automated SMS text message reminders were sent to patients after 2 weeks and 1 month, reading, “Hello, This is a reminder. It is essential that you contact your physician if you have not already done so.” As the SMS text messages were sent automatically to a randomly selected population, patients in the control group were not aware of the intervention. Physicians were blinded regarding which of their patients were in either group.

With no indication of whether a patient underwent colonoscopy, 120 days after the positive FOBT result, referring primary physicians (for both the control and intervention groups) answered a telephone survey regarding attitude toward FOBT as a screening test for CRC. We waited 120 days so as not to influence physicians’ interactions with their patients. We focused on whether FOBT is a reliable screening test for CRC and whether physicians recommend a repeat FOBT after obtaining a positive FOBT result, instead of follow-up, such as colonoscopy (Figure 1). Physicians who stated that FOBT is not a reliable screening test for CRC or reported that they would advise the patient to repeat FOBT instead of sending him or her for colonoscopy were considered as having a negative attitude toward FOBT.

### Statistical Analysis

Based on the statistical analysis of the Meuhedet patient database, we found that approximately 41% of patients with a positive FOBT result undergo colonoscopy within 120 days. To have an 80% chance of detecting a 20% increase in follow-up in the intervention group as significant (1-sided, 5% level), a sample of 77 was required in each group. The study sample of 232 in each group provided a 95% ability to detect a 20% increase in adherence to colonoscopy within 120 days.

Randomization was stratified by district and SES derived from members’ home address and based on the Israeli Census Bureau locality definitions [53]. SES levels ranged from 1 (low) to 20 (high). We compared demographic variables between the intervention and control groups using chi-square test for discrete variables. In addition, logistic regression was used to calculate odds ratios (ORs) and 95% CIs for the rate of colonoscopy within 120 days of receiving a positive FOBT result, adjusting for the main potential and variables found to be significantly related to adherence in univariate analysis. Data were analyzed using IBM SPSS Statistics for Windows (IBM Corp, 2016, Version 24.0). *P*<.05 was considered significant for all analyses.

## Results

### Study Population

Of 609 patients randomly selected from the patient database, 94 (15.4%) were excluded from the study sample: 46 (7.6%) because of family history of CRC, 22 (3.6%) because of an oncology diagnosis, and 26 (4.3%) because they had undergone colonoscopy prior to the positive FOBT result. A total of 470 eligible patients were randomized: 232 (49.4%) into the intervention group and 238 (50.6%) into the control group. Of them, 2 patients in the control group died during the study period and 43 (9.1%) in the intervention group refused to receive an SMS text message after their initial approval or were unable to receive SMS text message although they had agreed to receive it. Table 1 summarizes patient characteristics.

Just over half of the final participants were male (52.3%, 246/470), and the mean age of participants was 62.0 (SD 6.6) years. Most participants were from the South district, and the fewest were from the North district. In both groups, most participants were in SES levels 9-13, but this value was missing for 11.9% (56/470) patients. Gender rates were similar between the intervention and control groups, and the groups were ethnically similar. Furthermore, geographic dispersion was similar.

Overall, 163 of 232 patients in the intervention group and 112 of 238 patients in the control group underwent colonoscopy within 120 days of the positive FOBT result (70.3% vs 47.1%; *P*<.001). The unadjusted OR for completion of colonoscopy for the intervention versus control group was 2.17 (95% CI 1.49-3.17; *P*<.001).

Table 2 presents the bivariate (unadjusted) association between patient characteristics and adherence to colonoscopy within 120 days of a positive FOBT result for the entire cohort (N=470). Adherence rates were similar across genders. Adherence rates were higher in the Central and North districts than in the South and Jerusalem districts. Adherence rates were higher among those aged 50-59 and 70-75 years and among those with higher SES levels. Adherence rates to colonoscopy within 120 days of a positive FOBT result were higher among patients who had physicians with a positive attitude toward FOBT than among those who had physicians with a negative attitude toward FOBT (241/399, 60.4% vs 24/53, 45.3%; *P*=.04).

Logistic regression analysis was performed to determine whether colonoscopy rates differed between the groups after adjusting for potential confounders. The adjusted OR for adherence by the intervention group versus control group was 2.9 (95% CI 1.92-4.48, *P*=.001; Table 3). We performed the analysis including the 43 patients who did not receive all 3 SMS text messages (“intention to treat”) and found that adherence rates remained significantly higher both in the bivariate analysis (63.5% vs 47.1% in the control group; OR 1.96, 95% CI 1.37-2.79; *P*<.001) and in the multivariable model (OR 2.04, 95% CI 1.387-2.993; *P*<.001).

**Table 1:** Patient characteristics.

Characteristic		Control group (n=238), n (%)		Intervention group (n=232), n (%)		Total (N)	
**Gender**							
	Male		125 (52.5)		121 (52.2)		246
	Female		113 (47.5)		111 (47.8)		224
**Age (years)**							
	50-55		41 (17.2)		45 (19.5)		86
	55-59		40 (16.8)		39 (16.8)		79
	60-64		63 (26.5)		53 (22.8)		116
	65-69		66 (27.7)		66 (28.4)		132
	70-75		28 (11.8)		29 (12.5)		57
**Ethnicity**							
	Jewish		237 (99.6)		227 (97.8)		464
	Other		1 (0.4)		5 (2.2)		6
**National district**							
	South		83 (34.9)		99 (42.7)		182
	Jerusalem		60 (25.2)		49 (21.1)		109
	Center		50 (21.0)		46 (19.8)		96
	North		45 (18.9)		38 (16.4)		83
**Socioeconomic status level^a^**							
	1-8		13 (6.3)		20 (9.6)		33
	9-13		116 (56.6)		122 (58.4)		238
	14-20		76 (37.1)		67 (32.0)		143

^a^n=414; data missing for 56 patients.

**Table 2:** Adherence within 120 days after a positive fecal occult blood test result for individual variables.

Variable		Did not adhere to colonoscopy^a^ (n=195), n (%)	Adhered to colonoscopy^b^ (n=275), n (%)	*P* value
**Intervention versus control**				<.001
	Intervention	69 (29.7)	163 (70.3)	
	Control	126 (52.9)	112 (47.1)	
**Gender**				.26
	Male	96 (39)	150 (61)	
	Female	99 (44.2)	125 (55.8)	
**Age (years)**		195 (41.5)	275 (58.5)	.91
	50-54	35 (40.7)	51 (59.3)	
	55-59	32 (40.5)	47 (59.5)	
	60-64	49 (42.2)	67 (57.8)	
	65-69	56 (42.4)	76 (57.6)	
	70-75	23 (40.4)	34 (59.6)	
**District**		195 (41.5)	275 (58.5)	.36
	South	79 (43.4)	103 (56.6)	
	Jerusalem	51 (46.8)	58 (53.2)	
	Center	33 (34.4)	63 (65.6)	
	North	32 (38.6)	51 (61.4)	
**Socioeconomic status level^c^**				.10
	1-8	18 (54.5)	15 (45.5)	
	9-13	100 (42.0)	138 (58.0)	
	14-20	58 (40.6)	85 (59.4)	
**Physician attitude^d^**				.04
	Positive attitude	158 (39.6)	241 (60.4)	
	Negative attitude	29 (54.7)	24 (45.3)	

^a^Of all, 41.5% patients did not adhere to colonoscopy.

^b^Of all, 58.5% patients adhered to colonoscopy.

^c^Data missing for 56 patients.

^d^Data missing for 18 patients.

### Physicians

Among 282 primary physicians who referred participants to FOBT, 267 (94.7%) were interviewed, 4 (1.4%) refused, 9 (3.2%) could not be contacted, and 2 (0.9%) had left the organization. Most (83.5%, 223/267) physicians had a positive attitude toward FOBT as a tool for early detection of CRC. The adjusted OR for patients who had a physician with a positive attitude toward FOBT versus a negative attitude was 2.7 (95% CI 1.38-5.33; *P*=.004; Table 3). As some patients were referred for FOBT by the same primary care physician, we performed a binomial mixed model analysis to assess the impact of physician-level clustering. No difference was found, and the OR of the intervention group remained the same compared with that of the control group (2.9).

We conducted the intention-to-treat analysis including the 43 patients who did not receive the SMS text message and found that adherence rates remained significantly higher both in the bivariate (63.5% vs 47.1% in the control group; OR 1.96, 95% CI 1.37-2.79; *P*<.001) and the multivariable (OR 2.04, 95% CI 1.387-2.993; *P*<.001) analyses. As we only contacted physicians of patients who completed the study, we were unable to run the full multivariable model (including physician attitude).

**Table 3:** Multivariable analysis of colonoscopy rates.

Variable			Odds ratio		95% CI		*P* value
Intervention vs Control			2.93		1.92-4.48		<.001
Age (continuous)			1.01		0.98-1.04		.48
Female versus Male			0.81		0.53-1.22		.31
Socioeconomic status (continuous, 1-20)			0.99		0.92-1.07		.91
**South district**							
	Jerusalem district	1.26		0.72-2.20		.41	
	Center district	1.92		1.04-3.55		.04	
	North district	1.59		0.87-2.91		.13	
FOBT^a^ attitude: Positive versus Negative			2.72		1.38-5.33		.004
Constant			0.09		—^b^		.15

^a^FOBT: fecal occult blood test.

^b^Not applicable.

## Discussion

The effectiveness of mass cancer screening programs can be compromised by lack of follow-up of abnormal findings. Incomplete FOBT follow-up with colonoscopy is a significant problem that has been studied extensively. This study shows that sending SMS text message reminders to patients following a positive FOBT result is an effective way to increase adherence rates to follow-up colonoscopy. In this study, there was a relative increase of 49.2% in adherence in the intervention group using a simple, inexpensive means of communication. This surprisingly large increase in adherence could be a result of a combination of simplicity, repetition, and timeliness. Reminders were simply worded, with a clear message, sent immediately after results were obtained, and then repeated twice over the next month. Another potential advantage is that unlike telephone calls, which require active responses, the messages are “pushed.” Probably, patients who responded to the message were those who were more likely to complete the follow-up but required a “nudge.” Another possibility is that the message created cognitive dissonance in some patients who chose to ignore the results until they received the reminder. Future research should focus on how different contact methods are effective for different types of patients.

In this study, age, gender, SES, and geographic location were not significantly associated with adherence to follow-up of positive FOBT results either in the bivariate or multivariable analysis. Increasing age was previously associated with lack of follow-up in some studies [39], but not in others [21]. Similar to our findings, several studies did not find strong associations between gender and complete diagnostic follow-up [21,29,54]. However, others suggested that women are less likely than men to undergo follow-up testing [16,25,55]. Although the association between SES and follow-up was not significant, there appears to be a trend toward increasing follow-up rates at higher SES. This needs to be investigated further.

The physician plays an important role in a patient’s decision regarding follow-up tests. Trust in the physician and good communication between the patient and physician will positively influence the adherence rates of patients after a positive FOBT result. In this study, 95.1% (254/267) of physicians declared that FOBT is a reliable screening test for CRC and that they would not advise their patients to undergo FOBT again to ensure that the positive result is reliable. The physician’s attitude toward FOBT as a screening test significantly influences the patient’s adherence to colonoscopy.

A new finding of this study is that physicians’ attitude toward FOBT has a major influence on the rates of colonoscopy after a positive FOBT result. This finding may explain why intervention studies that provide physicians with knowledge and feedback have been effective. For example, Myers et al [56] showed that one-on-one physician training, audit, and feedback (physicians received lists of their patients with incomplete diagnostic evaluations) resulted in improved completion of diagnostic testing. Singh et al [57] assessed a clinic-based quality improvement activity that included provider education, a positive FOBT registry, and feedback; they found that it significantly decreased the time to colonoscopy referral and completion and increased colonoscopy completion by 18.7%. In spite of the fact that FOBT has been a standard practice for early detection of CRC, >10% of physicians in our study expressed mistrust in this method; this is possibly an underestimate, as some physicians may hesitate to express this view openly, which is contrary to organizational guidelines. It is, therefore, essential to educate physicians regarding the reliability and effectiveness of FOBT as a screening tool for CRC.

Owing to recent developments that have made digital communication within the health care sector readily available and inexpensive, SMS text message reminders could potentially be implemented in other areas. The finding of a large increase in follow-up after a positive FOBT result illustrates the need to further investigate different aspects of SMS text message usage, such as patients’ age, gender, the type of action they aim to increase, and the wording of the message. Changing technology and patient preferences with regard to contact communication should be considered when determining future interventions to improve usage and effectiveness.

A limitation of this study is that only patients who agreed to receive SMS text messages were included, which created a biased population in terms of age, cultural beliefs, and SES. An additional limitation is that SES was measured using zip code rather than a direct measure, and the SES of 56 participants was missing, although this is the standard method for measuring SES in Israel.

This study was conducted in 1 of the 4 health care provider organizations in Israel. Selection of providers was voluntary, and the member distribution in terms of age, gender, and SES was similar to that of the Israeli population. In addition, the Israeli population is very ethnically diverse and includes immigrants from many countries. Our findings are potentially generalizable to other populations.

In conclusion, this study is the first to directly evaluate SMS text message reminders for improving colonoscopy follow-up among Israeli CRC screening program participants following a positive FOBT result. We have shown that a simple, inexpensive intervention for patients improves colonoscopy follow-up after a positive FOBT result. It is important to maximize the potential of these findings by increasing the acceptance of SMS text messages within the population and to examine their use in other screening programs. In addition, it is important to examine, in future studies, the reasons because of which patients refuse to receive SMS text messages. The physician’s attitude toward FOBT as a screening test significantly influences patient adherence to colonoscopy. Therefore, further work needs to be done among physicians to increase adherence.

## Appendix

Multimedia Appendix 1 CONSORT‐EHEALTH checklist (V 1.6.1).

## Abbreviations

CRC: colorectal cancer
EMR: electronic medical record
FOBT: fecal occult blood test
OR: odds ratio
SES: socioeconomic status
SMS: short message service
