# Supplementary material for: Repeated Automated Mobile Text Messaging Reminders for Follow-Up of Positive Fecal Occult Blood Tests: Randomized Controlled Trial
Source: JMIR Mhealth Uhealth. 2019 Feb 5;7(2):e11114. doi: 10.2196/11114 (PMC6379817; doi:10.2196/11114)
Supplement: Multimedia Appendix 1 [file mhealth_v7i2e11114_app1.pdf]

CONSORT-EHEALTH (V 1.6.1) - Submission/Publication Form

The CONSORT-EHEALTH checklist is intended for authors of randomized trials evaluating web-based and Internet-based applications/interventions, including mobile interventions, electronic games (incl multiplayer games), social media, certain telehealth applications, and other interactive and/or networked electronic applications. Some of the items (e.g. all subitems under item 5 - description of the intervention) may also be applicable for other study designs.

The goal of the CONSORT EHEALTH checklist and guideline is to be
a) a guide for reporting for authors of RCTs,
b) to form a basis for appraisal of an ehealth trial (in terms of validity)

CONSORT-EHEALTH items/subitems are MANDATORY reporting items for studies published in the Journal of Medical Internet Research and other journals / scientific societies endorsing the checklist.

Items numbered 1., 2., 3., 4a., 4b etc are original CONSORT or CONSORT-NPT (non-pharmacologic treatment) items.
Items with Roman numerals (i., ii, iii, iv etc.) are CONSORT-EHEALTH extensions/clarifications.

As the CONSORT-EHEALTH checklist is still considered in a formative stage, we would ask that you also RATE ON A SCALE OF 1-5 how important/useful you feel each item is FOR THE PURPOSE OF THE CHECKLIST and reporting guideline (optional).

Mandatory reporting items are marked with a red *.
In the textboxes, either copy & paste the relevant sections from your manuscript into this form - please include any quotes from your manuscript in QUOTATION MARKS,
or answer directly by providing additional information not in the manuscript, or elaborating on why the item was not relevant for this study.

YOUR ANSWERS WILL BE PUBLISHED AS A SUPPLEMENTARY FILE TO YOUR PUBLICATION IN JMIR AND ARE CONSIDERED PART OF YOUR PUBLICATION (IF ACCEPTED).
Please fill in these questions diligently. Information will not be copyedited, so please use proper spelling and grammar, use correct capitalization, and avoid abbreviations.

DO NOT FORGET TO SAVE AS PDF _AND_ CLICK THE SUBMIT BUTTON SO YOUR ANSWERS ARE IN OUR DATABASE !!!

Citation Suggestion (if you append the pdf as Appendix we suggest to cite this paper in the caption):
Eysenbach G, CONSORT-EHEALTH Group
CONSORT-EHEALTH: Improving and Standardizing Evaluation Reports of Web-based and Mobile Health Interventions
J Med Internet Res 2011;13(4):e126
URL: http://www.jmir.org/2011/4/e126/
doi: 10.2196/jmir.1923
PMID: 22209829

בחרת לערוך את התשובה שלך. שיתוף כתובת האתר הזו יאפשר גם לאחרים לערוך את התשובה שלך.
הוספת תשובה חדשה

Your name *
First Last

REVITAL AZULAY

Primary Affiliation (short), City, Country *
University of Toronto, Toronto, Canada

MEUHEDET HEALTH FUND+BAR ILAN U

Your e-mail address *
abc@gmail.com

eazulay39@gmail.com

Title of your manuscript *
Provide the (draft) title of your manuscript.

Repeated automated mobile text reminders for follow-up of positive fecal occult blood tests - a randomised controlled trial

Name of your App/Software/Intervention *
If there is a short and a long/alternate name, write the short name first and add the long name in brackets.

SMS (telephone text messages)

Evaluated Version (if any)
e.g. "V1", "Release 2017-03-01", "Version 2.0.27913"

N/A

Language(s) *
What language is the intervention/app in? If multiple languages are available, separate by comma (e.g. "English, French")

בחרת לערוך את התשובה שלך. שיתוף כתובת האתר הזו יאפשר גם לאחרים לערוך את התשובה שלך.

הוספת תשובה חדשה

URL of your Intervention Website or App

e.g. a direct link to the mobile app on app in appstore (itunes, Google Play), or URL of the website. If the intervention is a DVD or hardware, you can also link to an Amazon page.

התשובה שלך

URL of an image/screenshot (optional)

התשובה שלך

Accessibility *

Can an enduser access the intervention presently?

- access is free and open
- access only for special usergroups, not open
- access is open to everyone, but requires payment/subscription/in-app purchases
- app/intervention no longer accessible
- (selected) אחר: Text messaging is a readily available technology and needs to be defin

Primary Medical Indication/Disease/Condition *

e.g. "Stress", "Diabetes", or define the target group in brackets after the condition, e.g. "Autism (Parents of children with)", "Alzheimers (Informal Caregivers of)"

Screening for clorectal cancer

Primary Outcomes measured in trial *

comma-separated list of primary outcomes reported in the trial

colonoscopy follow-up after a positive fe

Secondary/other outcomes

Are there any other outcomes the intervention is expected to affect?

בחרת לערוך את התשובה שלך. שיתוף כתובת האתר הזו יאפשר גם לאחרים לערוך את התשובה שלך.

הוספת תשובה חדשה

Recommended "Dose" *

What do the instructions for users say on how often the app should be used?

- ( ) Approximately Daily
- ( ) Approximately Weekly
- ( ) Approximately Monthly
- ( ) Approximately Yearly
- ( ) "as needed"
- (•) אחר: One week, a fortnight and a month after the positive test

Approx. Percentage of Users (starters) still using the app as recommended after 3 months *

- (•) unknown / not evaluated
- ( ) 0-10%
- ( ) 11-20%
- ( ) 21-30%
- ( ) 31-40%
- ( ) 41-50%
- ( ) 51-60%
- ( ) 61-70%
- ( ) 71%-80%
- ( ) 81-90%
- ( ) 91-100%
- ( ) אחר:

בחרת לערוך את התשובה שלך. שיתוף כתובת האתר הזו יאפשר גם לאחרים לערוך את התשובה שלך.

הוספת תשובה חדשה

Overall, was the app/intervention effective? *

- (selected) yes: all primary outcomes were significantly better in intervention group vs control
- partly: SOME primary outcomes were significantly better in intervention group vs control
- no statistically significant difference between control and intervention
- potentially harmful: control was significantly better than intervention in one or more outcomes
- inconclusive: more research is needed
- אחר:

Article Preparation Status/Stage *

At which stage in your article preparation are you currently (at the time you fill in this form)

- not submitted yet - in early draft status
- not submitted yet - in late draft status, just before submission
- submitted to a journal but not reviewed yet
- (selected) submitted to a journal and after receiving initial reviewer comments
- submitted to a journal and accepted, but not published yet
- published
- אחר:

בחרת לערוך את התשובה שלך. שיתוף כתובת האתר הזו יאפשר גם לאחרים לערוך את התשובה שלך.

הוספת תשובה חדשה

Journal *

If you already know where you will submit this paper (or if it is already submitted), please provide the journal name (if it is not JMIR, provide the journal name under "other")

- ○ not submitted yet / unclear where I will submit this
- ○ Journal of Medical Internet Research (JMIR)
- ◉ JMIR mHealth and UHealth
- ○ JMIR Serious Games
- ○ JMIR Mental Health
- ○ JMIR Public Health
- ○ JMIR Formative Research
- ○ Other JMIR sister journal
- ○ אחר:

Is this a full powered effectiveness trial or a pilot/feasibility trial? *

- ○ Pilot/feasibility
- ◉ Fully powered

Manuscript tracking number *

If this is a JMIR submission, please provide the manuscript tracking number under "other" (The ms tracking number can be found in the submission acknowledgement email, or when you login as author in JMIR. If the paper is already published in JMIR, then the ms tracking number is the four-digit number at the end of the DOI, to be found at the bottom of each published article in JMIR)

- ○ no ms number (yet) / not (yet) submitted to / published in JMIR
- ◉ אחר: JMU11114

TITLE AND ABSTRACT

בחרת לערוך את התשובה שלך. שיתוף כתובת האתר הזו יאפשר גם לאחרים לערוך את התשובה שלך.

הוספת תשובה חדשה

1a) Does your paper address CONSORT item 1a? *

I.e does the title contain the phrase "Randomized Controlled Trial"? (if not, explain the reason under "other")

- (●) yes
- ( ) :אחר

1a-i) Identify the mode of delivery in the title

Identify the mode of delivery. Preferably use “web-based” and/or “mobile” and/or “electronic game” in the title. Avoid ambiguous terms like “online”, “virtual”, “interactive”. Use “Internet-based” only if Intervention includes non-web-based Internet components (e.g. email), use “computer-based” or “electronic” only if offline products are used. Use “virtual” only in the context of “virtual reality” (3-D worlds). Use “online” only in the context of “online support groups”. Complement or substitute product names with broader terms for the class of products (such as “mobile” or “smart phone” instead of “iphone”), especially if the application runs on different platforms.

	5	4	3	2	1	
essential	(●)	( )	( )	( )	( )	subitem not at all important

Does your paper address subitem 1a-i? *

Copy and paste relevant sections from manuscript title (include quotes in quotation marks "like this" to indicate direct quotes from your manuscript), or elaborate on this item by providing additional information not in the ms, or briefly explain why the item is not applicable/relevant for your study

Yes-Repeated automated mobile text reminders for follow-up of positive fecal occult blood tests - a randomised controlled trial

1a-ii) Non-web-based components or important co-interventions in title

Mention non-web-based components or important co-interventions in title, if any (e.g., “with telephone support”).

	5	4	3	2	1	
essential	( )	( )	( )	( )	(●)	subitem not at all important

Does your paper address subitem 1a-ii?

Copy and paste relevant sections from manuscript title (include quotes in quotation marks "like this" to indicate direct quotes from your manuscript), or elaborate on this item by providing additional information not in the ms, or briefly explain why the item is not applicable/relevant for your study

בחרת לערוך את התשובה שלך. שיתוף כתובת האתר הזו יאפשר גם לאחרים לערוך את התשובה שלך.

הוספת תשובה חדשה

1a-iii) Primary condition or target group in the title

Mention primary condition or target group in the title, if any (e.g., “for children with Type I Diabetes”) Example: A Web-based and Mobile Intervention with Telephone Support for Children with Type I Diabetes: Randomized Controlled Trial

	5	4	3	2	1	
essential	◉	○	○	○	○	subitem not at all important

Does your paper address subitem 1a-iii? *

Copy and paste relevant sections from manuscript title (include quotes in quotation marks "like this" to indicate direct quotes from your manuscript), or elaborate on this item by providing additional information not in the ms, or briefly explain why the item is not applicable/relevant for your study

Yes-Repeated automated mobile text reminders for follow-up of positive fecal occult blood tests - a randomised controlled trial

1b) ABSTRACT: Structured summary of trial design, methods, results, and conclusions

NPT extension: Description of experimental treatment, comparator, care providers, centers, .and blinding status

1b-i) Key features/functionalities/components of the intervention and comparator in the METHODS section of the ABSTRACT

Mention key features/functionalities/components of the intervention and comparator in the abstract. If possible, also mention theories and principles used for designing the site. Keep in mind the needs of systematic reviewers and indexers by including important synonyms. (Note: Only report in the abstract what the main paper is reporting. If this information is missing from the main body of text, consider adding it)

	5	4	3	2	1	
essential	◉	○	○	○	○	subitem not at all important

Does your paper address subitem 1b-i? *

Copy and paste relevant sections from the manuscript abstract (include quotes in quotation marks "like this" to indicate direct quotes from your manuscript), or elaborate on this item by providing additional information not in the ms, or briefly explain why the item is not applicable/relevant for your study

Yes- "The intervention group (n = 232) received routine care and three short

בחרת לערוך את התשובה שלך. שיתוף כתובת האתר הזו יאפשר גם לאחרים לערוך את התשובה שלך.

הוספת תשובה חדשה

1b-ii) Level of human involvement in the METHODS section of the ABSTRACT

Clarify the level of human involvement in the abstract, e.g., use phrases like “fully automated” vs. “therapist/nurse/care provider/physician-assisted” (mention number and expertise of providers involved, if any). (Note: Only report in the abstract what the main paper is reporting. If this information is missing from the main body of text, consider adding it)

	5	4	3	2	1	
essential	○	○	○	○	◉	subitem not at all important

Does your paper address subitem 1b-ii?

Copy and paste relevant sections from the manuscript abstract (include quotes in quotation marks "like this" to indicate direct quotes from your manuscript), or elaborate on this item by providing additional information not in the ms, or briefly explain why the item is not applicable/relevant for your study

No human involvement necessary -fully automated

1b-iii) Open vs. closed, web-based (self-assessment) vs. face-to-face assessments in the METHODS section of the ABSTRACT

Mention how participants were recruited (online vs. offline), e.g., from an open access website or from a clinic or a closed online user group (closed usergroup trial), and clarify if this was a purely web-based trial, or there were face-to-face components (as part of the intervention or for assessment). Clearly say if outcomes were self-assessed through questionnaires (as common in web-based trials). Note: In traditional offline trials, an open trial (open-label trial) is a type of clinical trial in which both the researchers and participants know which treatment is being administered. To avoid confusion, use “blinded” or “unblinded” to indicated the level of blinding instead of “open”, as “open” in web-based trials usually refers to “open access” (i.e. participants can self-enrol). (Note: Only report in the abstract what the main paper is reporting. If this information is missing from the main body of text, consider adding it)

	5	4	3	2	1	
essential	○	◉	○	○	○	subitem not at all important

Does your paper address subitem 1b-iii?

Copy and paste relevant sections from the manuscript abstract (include quotes in quotation marks "like this" to indicate direct quotes from your manuscript), or elaborate on this item by providing additional information not in the ms, or briefly explain why the item is not applicable/relevant for your study

"This randomized controlled trial was conducted with patients who had positive colorectal cancer screening results.". and "All patient information, including test [illegible]

בחרת לערוך את התשובה שלך. שיתוף כתובת האתר הזו יאפשר גם לאחרים לערוך את התשובה שלך.

הוספת תשובה חדשה

1b-iv) RESULTS section in abstract must contain use data

Report number of participants enrolled/assessed in each group, the use/uptake of the intervention (e.g., attrition/adherence metrics, use over time, number of logins etc.), in addition to primary/secondary outcomes. (Note: Only report in the abstract what the main paper is reporting. If this information is missing from the main body of text, consider adding it)

	5	4	3	2	1	
essential	○	○	○	○	◉	subitem not at all important

Does your paper address subitem 1b-iv?

Copy and paste relevant sections from the manuscript abstract (include quotes in quotation marks "like this" to indicate direct quotes from your manuscript), or elaborate on this item by providing additional information not in the ms, or briefly explain why the item is not applicable/relevant for your study

As the intervention was passive (reception of text messages) we addressed this in the methods: "The intervention group (n = 232) received routine care and three short message service reminders to visit their primary care physician"..."Results: Of 232 patients in the intervention group, 163 had a colonoscopy within 120 days from the positive FOBT, and of 238 patients in the control group, 112 of 238 had a colonoscopy within 120 days from the positive FOBT (70% vs. 47%, P<.0001). "

1b-v) CONCLUSIONS/DISCUSSION in abstract for negative trials

Conclusions/Discussions in abstract for negative trials: Discuss the primary outcome - if the trial is negative (primary outcome not changed), and the intervention was not used, discuss whether negative results are attributable to lack of uptake and discuss reasons. (Note: Only report in the abstract what the main paper is reporting. If this information is missing from the main body of text, consider adding it)

	5	4	3	2	1	
essential	○	○	○	○	◉	subitem not at all important

Does your paper address subitem 1b-v?

Copy and paste relevant sections from the manuscript abstract (include quotes in quotation marks "like this" to indicate direct quotes from your manuscript), or elaborate on this item by providing additional information not in the ms, or briefly explain why the item is not applicable/relevant for your study

N/A

בחרת לערוך את התשובה שלך. שיתוף כתובת האתר הזו יאפשר גם לאחרים לערוך את התשובה שלך.

הוספת תשובה חדשה

2a) In INTRODUCTION: Scientific background and explanation of rationale

2a-i) Problem and the type of system/solution

Describe the problem and the type of system/solution that is object of the study: intended as stand-alone intervention vs. incorporated in broader health care program? Intended for a particular patient population? Goals of the intervention, e.g., being more cost-effective to other interventions, replace or complement other solutions? (Note: Details about the intervention are provided in “Methods” under 5)

	5	4	3	2	1	
essential	(selected)	○	○	○	○	subitem not at all important

Does your paper address subitem 2a-i? *

Copy and paste relevant sections from the manuscript (include quotes in quotation marks "like this" to indicate direct quotes from your manuscript), or elaborate on this item by providing additional information not in the ms, or briefly explain why the item is not applicable/relevant for your study

"no studies have yet used SMS technology to increase adherence to recommendations for CRC screening follow-up after a positive FOBT."

2a-ii) Scientific background, rationale: What is known about the (type of) system

Scientific background, rationale: What is known about the (type of) system that is the object of the study (be sure to discuss the use of similar systems for other conditions/diagnoses, if appropiate), motivation for the study, i.e. what are the reasons for and what is the context for this specific study, from which stakeholder viewpoint is the study performed, potential impact of findings [2]. Briefly justify the choice of the comparator.

	5	4	3	2	1	
essential	○	(selected)	○	○	○	subitem not at all important

בחרת לערוך את התשובה שלך. שיתוף כתובת האתר הזו יאפשר גם לאחרים לערוך את התשובה שלך.

הוספת תשובה חדשה

Does your paper address subitem 2a-ii? *

Copy and paste relevant sections from the manuscript (include quotes in quotation marks "like this" to indicate direct quotes from your manuscript), or elaborate on this item by providing additional information not in the ms, or briefly explain why the item is not applicable/relevant for your study

"A positive FOBT requires immediate follow-up with colonoscopy and surgery should be performed when CRC is found. .A delay in follow-up significantly undermines the benefits of CRC screening, including incidence, mortality, life-years saved, and net costs of screening ", "Several studies have described interventions aimed at increasing follow-up rates and showed mixed results", ""no studies have yet used SMS technology to increase adherence to recommendations for CRC screening follow-up after a positive FOBT."

2b) In INTRODUCTION: Specific objectives or hypotheses

Does your paper address CONSORT subitem 2b? *

Copy and paste relevant sections from the manuscript (include quotes in quotation marks "like this" to indicate direct quotes from your manuscript), or elaborate on this item by providing additional information not in the ms, or briefly explain why the item is not applicable/relevant for your study

"the present study examined the effectiveness of sending mobile text messages via SMS to patients as an automated tool to increase adherence to colonoscopy follow-up"

METHODS

3a) Description of trial design (such as parallel, factorial) including allocation ratio

Does your paper address CONSORT subitem 3a? *

Copy and paste relevant sections from the manuscript (include quotes in quotation marks "like this" to indicate direct quotes from your manuscript), or elaborate on this item by providing additional information not in the ms, or briefly explain why the item is not applicable/relevant for your study

Figure 1 (attached)

3b) Important changes to methods after trial commencement

בחרת לערוך את התשובה שלך. שיתוף כתובת האתר הזו יאפשר גם לאחרים לערוך את התשובה שלך.

הוספת תשובה חדשה

Does your paper address CONSORT subitem 3b? *

Copy and paste relevant sections from the manuscript (include quotes in quotation marks "like this" to indicate direct quotes from your manuscript), or elaborate on this item by providing additional information not in the ms, or briefly explain why the item is not applicable/relevant for your study

Methods did not change

3b-i) Bug fixes, Downtimes, Content Changes

Bug fixes, Downtimes, Content Changes: ehealth systems are often dynamic systems. A description of changes to methods therefore also includes important changes made on the intervention or comparator during the trial (e.g., major bug fixes or changes in the functionality or content) (5-iii) and other “unexpected events” that may have influenced study design such as staff changes, system failures/downtimes, etc. [2].

	5	4	3	2	1	
essential	○	○	○	○	◉	subitem not at all important

Does your paper address subitem 3b-i?

Copy and paste relevant sections from the manuscript (include quotes in quotation marks "like this" to indicate direct quotes from your manuscript), or elaborate on this item by providing additional information not in the ms, or briefly explain why the item is not applicable/relevant for your study

This is a simple technology and no bugs or downtimes occurred

4a) Eligibility criteria for participants

Does your paper address CONSORT subitem 4a? *

Copy and paste relevant sections from the manuscript (include quotes in quotation marks "like this" to indicate direct quotes from your manuscript), or elaborate on this item by providing additional information not in the ms, or briefly explain why the item is not applicable/relevant for your study

"The inclusion criteria were 50-75 years-of-age and agreeing to receive SMS texts from Meuhedet. By Israeli law, health messages via SMS may only be sent to members who actively agree to receive them."

4a-i) Computer / Internet literacy

Computer / Internet literacy is often an implicit “de facto” eligibility criterion - this should be explicitly clarified.

5	4	3	2	1

בחרת לערוך את התשובה שלך. שיתוף כתובת האתר הזו יאפשר גם לאחרים לערוך את התשובה שלך.
הוספת תשובה חדשה

Does your paper address subitem 4a-i?

Copy and paste relevant sections from the manuscript (include quotes in quotation marks "like this" to indicate direct quotes from your manuscript), or elaborate on this item by providing additional information not in the ms, or briefly explain why the item is not applicable/relevant for your study

"The inclusion criteria were 50-75 years-of-age and agreeing to receive SMS texts from Meuhedet. By Israeli law, health messages via SMS may only be sent to members who actively agree to receive them."

4a-ii) Open vs. closed, web-based vs. face-to-face assessments:

Open vs. closed, web-based vs. face-to-face assessments: Mention how participants were recruited (online vs. offline), e.g., from an open access website or from a clinic, and clarify if this was a purely web-based trial, or there were face-to-face components (as part of the intervention or for assessment), i.e., to what degree got the study team to know the participant. In online-only trials, clarify if participants were quasi-anonymous and whether having multiple identities was possible or whether technical or logistical measures (e.g., cookies, email confirmation, phone calls) were used to detect/prevent these.

	5	4	3	2	1	
essential	○	○	○	○	●	subitem not at all important

Does your paper address subitem 4a-ii? *

Copy and paste relevant sections from the manuscript (include quotes in quotation marks "like this" to indicate direct quotes from your manuscript), or elaborate on this item by providing additional information not in the ms, or briefly explain why the item is not applicable/relevant for your study

All patients were recruited from our patient database and all patient information was obtained from the same database

4a-iii) Information giving during recruitment

Information given during recruitment. Specify how participants were briefed for recruitment and in the informed consent procedures (e.g., publish the informed consent documentation as appendix, see also item X26), as this information may have an effect on user self-selection, user expectation and may also bias results.

	5	4	3	2	1	
essential	○	○	○	○	●	subitem not at all important

בחרת לערוך את התשובה שלך. שיתוף כתובת האתר הזו יאפשר גם לאחרים לערוך את התשובה שלך.

הוספת תשובה חדשה

Does your paper address subitem 4a-iii?

Copy and paste relevant sections from the manuscript (include quotes in quotation marks "like this" to indicate direct quotes from your manuscript), or elaborate on this item by providing additional information not in the ms, or briefly explain why the item is not applicable/relevant for your study

All patients were recruited from our patient database and all patient information was obtained from the same database

4b) Settings and locations where the data were collected

Does your paper address CONSORT subitem 4b? *

Copy and paste relevant sections from the manuscript (include quotes in quotation marks "like this" to indicate direct quotes from your manuscript), or elaborate on this item by providing additional information not in the ms, or briefly explain why the item is not applicable/relevant for your study

All patients were recruited from our patient database and all patient information was obtained from the same database

4b-i) Report if outcomes were (self-)assessed through online questionnaires

Clearly report if outcomes were (self-)assessed through online questionnaires (as common in web-based trials) or otherwise.

	5	4	3	2	1	
essential	○	○	○	○	◉	subitem not at all important

Does your paper address subitem 4b-i? *

Copy and paste relevant sections from the manuscript (include quotes in quotation marks "like this" to indicate direct quotes from your manuscript), or elaborate on this item by providing additional information not in the ms, or briefly explain why the item is not applicable/relevant for your study

All patients were recruited from our patient database and all patient information, including outcome, was obtained from the same database

4b-ii) Report how institutional affiliations are displayed

Report how institutional affiliations are displayed to potential participants [on ehealth media], as affiliations with prestigious hospitals or universities may affect volunteer rates, use, and reactions with regards to an intervention.(Not a required item – describe only if this may bias results)

5 4 3 2 1

בחרת לערוך את התשובה שלך. שיתוף כתובת האתר הזו יאפשר גם לאחרים לערוך את התשובה שלך.

הוספת תשובה חדשה

Does your paper address subitem 4b-ii?

Copy and paste relevant sections from the manuscript (include quotes in quotation marks "like this" to indicate direct quotes from your manuscript), or elaborate on this item by providing additional information not in the ms, or briefly explain why the item is not applicable/relevant for your study

All patients were recruited from our patient database and all patient information was obtained from the same database

The interventions for each group with sufficient details to allow replication, including how and when they were actually administered (5

5-i) Mention names, credential, affiliations of the developers, sponsors, and owners

Mention names, credential, affiliations of the developers, sponsors, and owners [6] (if authors/evaluators are owners or developer of the software, this needs to be declared in a “Conflict of interest” section or mentioned elsewhere in the manuscript).

	5	4	3	2	1	
essential	○	○	○	○	◉	subitem not at all important

Does your paper address subitem 5-i?

Copy and paste relevant sections from the manuscript (include quotes in quotation marks "like this" to indicate direct quotes from your manuscript), or elaborate on this item by providing additional information not in the ms, or briefly explain why the item is not applicable/relevant for your study

Only text messages were used.

5-ii) Describe the history/development process

Describe the history/development process of the application and previous formative evaluations (e.g., focus groups, usability testing), as these will have an impact on adoption/use rates and help with interpreting results.

	5	4	3	2	1	
essential	○	○	○	○	◉	subitem not at all important

בחרת לערוך את התשובה שלך. שיתוף כתובת האתר הזו יאפשר גם לאחרים לערוך את התשובה שלך.
הוספת תשובה חדשה

Does your paper address subitem 5-ii?

Copy and paste relevant sections from the manuscript (include quotes in quotation marks "like this" to indicate direct quotes from your manuscript), or elaborate on this item by providing additional information not in the ms, or briefly explain why the item is not applicable/relevant for your study

Only text messages were used

5-iii) Revisions and updating

Revisions and updating. Clearly mention the date and/or version number of the application/intervention (and comparator, if applicable) evaluated, or describe whether the intervention underwent major changes during the evaluation process, or whether the development and/or content was “frozen” during the trial. Describe dynamic components such as news feeds or changing content which may have an impact on the replicability of the intervention (for unexpected events see item 3b).

	5	4	3	2	1	
essential	○	○	○	○	◉	subitem not at all important

Does your paper address subitem 5-iii?

Copy and paste relevant sections from the manuscript (include quotes in quotation marks "like this" to indicate direct quotes from your manuscript), or elaborate on this item by providing additional information not in the ms, or briefly explain why the item is not applicable/relevant for your study

No revisions or updating

5-iv) Quality assurance methods

Provide information on quality assurance methods to ensure accuracy and quality of information provided [1], if applicable.

	5	4	3	2	1	
essential	○	○	◉	○	○	subitem not at all important

בחרת לערוך את התשובה שלך. שיתוף כתובת האתר הזו יאפשר גם לאחרים לערוך את התשובה שלך.

הוספת תשובה חדשה

Does your paper address subitem 5-iv?

Copy and paste relevant sections from the manuscript (include quotes in quotation marks "like this" to indicate direct quotes from your manuscript), or elaborate on this item by providing additional information not in the ms, or briefly explain why the item is not applicable/relevant for your study

"Each patient in the intervention group also received an automated text message up to a week after the positive FOBT result. The text read: "Hello. There is a lab test result ready for you. Contact your physician for an explanation of the findings." Two additional automated text message reminders were sent to the patient at 2 weeks and 1 month reading, "Hello, This is a reminder. It is essential that you contact your physician if you have not already done so." As the SMS messages were sent automatically to a randomly selected population, the patients in the control group were not aware of the intervention. Physicians were blinded regarding which of their patients were in either group."

5-v) Ensure replicability by publishing the source code, and/or providing screenshots/screen-capture video, and/or providing flowcharts of the algorithms used

Ensure replicability by publishing the source code, and/or providing screenshots/screen-capture video, and/or providing flowcharts of the algorithms used. Replicability (i.e., other researchers should in principle be able to replicate the study) is a hallmark of scientific reporting.

	5	4	3	2	1	
essential	○	○	○	○	◉	subitem not at all important

Does your paper address subitem 5-v?

Copy and paste relevant sections from the manuscript (include quotes in quotation marks "like this" to indicate direct quotes from your manuscript), or elaborate on this item by providing additional information not in the ms, or briefly explain why the item is not applicable/relevant for your study

N/A

5-vi) Digital preservation

Digital preservation: Provide the URL of the application, but as the intervention is likely to change or disappear over the course of the years; also make sure the intervention is archived (Internet Archive, webcitation.org, and/or publishing the source code or screenshots/videos alongside the article). As pages behind login screens cannot be archived, consider creating demo pages which are accessible without login.

5	4	3	2	1

בחרת לערוך את התשובה שלך. שיתוף כתובת האתר הזו יאפשר גם לאחרים לערוך את התשובה שלך.
הוספת תשובה חדשה

Does your paper address subitem 5-vi?

Copy and paste relevant sections from the manuscript (include quotes in quotation marks "like this" to indicate direct quotes from your manuscript), or elaborate on this item by providing additional information not in the ms, or briefly explain why the item is not applicable/relevant for your study

N/A

5-vii) Access

Access: Describe how participants accessed the application, in what setting/context, if they had to pay (or were paid) or not, whether they had to be a member of specific group. If known, describe how participants obtained “access to the platform and Internet” [1]. To ensure access for editors/reviewers/readers, consider to provide a “backdoor” login account or demo mode for reviewers/readers to explore the application (also important for archiving purposes, see vi).

	5	4	3	2	1	
essential	○	◉	○	○	○	subitem not at all important

Does your paper address subitem 5-vii? *

Copy and paste relevant sections from the manuscript (include quotes in quotation marks "like this" to indicate direct quotes from your manuscript), or elaborate on this item by providing additional information not in the ms, or briefly explain why the item is not applicable/relevant for your study

"Each patient in the intervention group also received an automated text message up to a week after the positive FOBT result. The text read: "Hello. There is a lab test result ready for you. Contact your physician for an explanation of the findings." Two additional automated text message reminders were sent to the patient at 2 weeks and 1 month reading, "Hello, This is a reminder. It is essential that you contact your physician if you have not already done so." As the SMS messages were sent automatically to a randomly selected population, the patients in the control group were not aware of the intervention. Physicians were blinded regarding which of their patients were in either group."

הוספת תשובה חדשה

בחרת לערוך את התשובה שלך. שיתוף כתובת האתר הזו יאפשר גם לאחרים לערוך את התשובה שלך.

5-viii) Mode of delivery, features/functionalities/components of the intervention and comparator, and the theoretical framework

Describe mode of delivery, features/functionalities/components of the intervention and comparator, and the theoretical framework [6] used to design them (instructional strategy [1], behaviour change techniques, persuasive features, etc., see e.g., [7, 8] for terminology). This includes an in-depth description of the content (including where it is coming from and who developed it) [1]," whether [and how] it is tailored to individual circumstances and allows users to track their progress and receive feedback" [6]. This also includes a description of communication delivery channels and – if computer-mediated communication is a component – whether communication was synchronous or asynchronous [6]. It also includes information on presentation strategies [1], including page design principles, average amount of text on pages, presence of hyperlinks to other resources, etc. [1].

	5	4	3	2	1	
essential	○	○	○	○	◉	subitem not at all important

Does your paper address subitem 5-viii? *

Copy and paste relevant sections from the manuscript (include quotes in quotation marks "like this" to indicate direct quotes from your manuscript), or elaborate on this item by providing additional information not in the ms, or briefly explain why the item is not applicable/relevant for your study

N/A

5-ix) Describe use parameters

Describe use parameters (e.g., intended "doses" and optimal timing for use). Clarify what instructions or recommendations were given to the user, e.g., regarding timing, frequency, heaviness of use, if any, or was the intervention used ad libitum.

	5	4	3	2	1	
essential	◉	○	○	○	○	subitem not at all important

Does your paper address subitem 5-ix?

Copy and paste relevant sections from the manuscript (include quotes in quotation marks "like this" to indicate direct quotes from your manuscript), or elaborate on this item by providing additional information not in the ms, or briefly explain why the item is not applicable/relevant for your study

"Each patient in the intervention group also received an automated text message up to a week after the positive FOBT result. The text read: "Hello. There is a lab test result ready for you. Contact your physician for an explanation of the findings." Two additional automated text message reminders were sent to the patient at 2 weeks and 1 month reading, "Hello, This is a reminder. It is essential that you contact your physician if you have not already done so." ."

בחרת לערוך את התשובה שלך. שיתוף כתובת האתר הזו יאפשר גם לאחרים לערוך את התשובה שלך.
הוספת תשובה חדשה

5-x) Clarify the level of human involvement

Clarify the level of human involvement (care providers or health professionals, also technical assistance) in the e-intervention or as co-intervention (detail number and expertise of professionals involved, if any, as well as "type of assistance offered, the timing and frequency of the support, how it is initiated, and the medium by which the assistance is delivered". It may be necessary to distinguish between the level of human involvement required for the trial, and the level of human involvement required for a routine application outside of a RCT setting (discuss under item 21 – generalizability).

	5	4	3	2	1	
essential	○	○	○	○	◉	subitem not at all important

Does your paper address subitem 5-x?

Copy and paste relevant sections from the manuscript (include quotes in quotation marks "like this" to indicate direct quotes from your manuscript), or elaborate on this item by providing additional information not in the ms, or briefly explain why the item is not applicable/relevant for your study

Fully automated

5-xi) Report any prompts/reminders used

Report any prompts/reminders used: Clarify if there were prompts (letters, emails, phone calls, SMS) to use the application, what triggered them, frequency etc. It may be necessary to distinguish between the level of prompts/reminders required for the trial, and the level of prompts/reminders for a routine application outside of a RCT setting (discuss under item 21 – generalizability).

	5	4	3	2	1	
essential	◉	○	○	○	○	subitem not at all important

Does your paper address subitem 5-xi? *

Copy and paste relevant sections from the manuscript (include quotes in quotation marks "like this" to indicate direct quotes from your manuscript), or elaborate on this item by providing additional information not in the ms, or briefly explain why the item is not applicable/relevant for your study

"Each patient in the intervention group also received an automated text message up to a week after the positive FOBT result. The text read: "Hello. There is a lab test result ready for you. Contact your physician for an explanation of the findings." Two additional automated text message reminders were sent to the patient at 2 weeks and 1 month reading, "Hello, This is a reminder. It is essential that you contact your physician if you have not already done so." ."

בחרת לערוך את התשובה שלך. שיתוף כתובת האתר הזו יאפשר גם לאחרים לערוך את התשובה שלך.

הוספת תשובה חדשה

5-xii) Describe any co-interventions (incl. training/support)

Describe any co-interventions (incl. training/support): Clearly state any interventions that are provided in addition to the targeted eHealth intervention, as ehealth intervention may not be designed as stand-alone intervention. This includes training sessions and support [1]. It may be necessary to distinguish between the level of training required for the trial, and the level of training for a routine application outside of a RCT setting (discuss under item 21 – generalizability.

	5	4	3	2	1	
essential	○	○	○	○	◉	subitem not at all important

Does your paper address subitem 5-xii? *

Copy and paste relevant sections from the manuscript (include quotes in quotation marks "like this" to indicate direct quotes from your manuscript), or elaborate on this item by providing additional information not in the ms, or briefly explain why the item is not applicable/relevant for your study

None

6a) Completely defined pre-specified primary and secondary outcome measures, including how and when they were assessed

Does your paper address CONSORT subitem 6a? *

Copy and paste relevant sections from the manuscript (include quotes in quotation marks "like this" to indicate direct quotes from your manuscript), or elaborate on this item by providing additional information not in the ms, or briefly explain why the item is not applicable/relevant for your study

"The dependent variable was adherence to colonoscopy within 120 days after a positive FOBT. This was obtained from the patient's EMR."

6a-i) Online questionnaires: describe if they were validated for online use and apply CHERRIES items to describe how the questionnaires were designed/deployed

If outcomes were obtained through online questionnaires, describe if they were validated for online use and apply CHERRIES items to describe how the questionnaires were designed/deployed [9].

	5	4	3	2	1	
essential	○	○	○	○	◉	subitem not at all important

בחרת לערוך את התשובה שלך. שיתוף כתובת האתר הזו יאפשר גם לאחרים לערוך את התשובה שלך.

הוספת תשובה חדשה

Does your paper address subitem 6a-i?

Copy and paste relevant sections from manuscript text

N/A

6a-ii) Describe whether and how “use” (including intensity of use/dosage) was defined/measured/monitored

Describe whether and how “use” (including intensity of use/dosage) was defined/measured/monitored (logins, logfile analysis, etc.). Use/adoption metrics are important process outcomes that should be reported in any ehealth trial.

	5	4	3	2	1	
essential	◉	○	○	○	○	subitem not at all important

Does your paper address subitem 6a-ii?

Copy and paste relevant sections from manuscript text

"Each patient in the intervention group also received an automated text message up to a week after the positive FOBT result. The text read: "Hello. There is a lab test result ready for you. Contact your physician for an explanation of the findings." Two additional automated text message reminders were sent to the patient at 2 weeks and 1 month reading, "Hello, This is a reminder. It is essential that you contact your physician if you have not already done so."."

6a-iii) Describe whether, how, and when qualitative feedback from participants was obtained

Describe whether, how, and when qualitative feedback from participants was obtained (e.g., through emails, feedback forms, interviews, focus groups).

	5	4	3	2	1	
essential	○	○	○	○	◉	subitem not at all important

Does your paper address subitem 6a-iii?

Copy and paste relevant sections from manuscript text

N/A

הוספת תשובה חדשה

בחרת לערוך את התשובה שלך. שיתוף כתובת האתר הזו יאפשר גם לאחרים לערוך את התשובה שלך.

6b) Any changes to trial outcomes after the trial commenced, with reasons

Does your paper address CONSORT subitem 6b? *

Copy and paste relevant sections from the manuscript (include quotes in quotation marks "like this" to indicate direct quotes from your manuscript), or elaborate on this item by providing additional information not in the ms, or briefly explain why the item is not applicable/relevant for your study

No changes were made

7a) How sample size was determined

NPT: When applicable, details of whether and how the clustering by care provides or centers was addressed

7a-i) Describe whether and how expected attrition was taken into account when calculating the sample size

Describe whether and how expected attrition was taken into account when calculating the sample size.

	5	4	3	2	1	
essential	(●)	( )	( )	( )	( )	subitem not at all important

Does your paper address subitem 7a-i?

Copy and paste relevant sections from manuscript title (include quotes in quotation marks "like this" to indicate direct quotes from your manuscript), or elaborate on this item by providing additional information not in the ms, or briefly explain why the item is not applicable/relevant for your study

"To have an 80% chance of detecting a 20% effect of the intervention as significant (1-sided, 5% level), a sample of 77 is required in each group. Our group was larger than this.. Our study sample of 232 in each group has a 95% ability to detect a 20% increase in adherence to colonoscopy within 120 days."

7b) When applicable, explanation of any interim analyses and stopping guidelines

בחרת לערוך את התשובה שלך. שיתוף כתובת האתר הזו יאפשר גם לאחרים לערוך את התשובה שלך.

הוספת תשובה חדשה

Does your paper address CONSORT subitem 7b? *

Copy and paste relevant sections from the manuscript (include quotes in quotation marks "like this" to indicate direct quotes from your manuscript), or elaborate on this item by providing additional information not in the ms, or briefly explain why the item is not applicable/relevant for your study

N/A

8a) Method used to generate the random allocation sequence

NPT: When applicable, how care providers were allocated to each trial group

Does your paper address CONSORT subitem 8a? *

Copy and paste relevant sections from the manuscript (include quotes in quotation marks "like this" to indicate direct quotes from your manuscript), or elaborate on this item by providing additional information not in the ms, or briefly explain why the item is not applicable/relevant for your study

Patients were randomised electronically as all information is on a database, including intervention.

8b) Type of randomisation; details of any restriction (such as (blocking and block size

Does your paper address CONSORT subitem 8b? *

Copy and paste relevant sections from the manuscript (include quotes in quotation marks "like this" to indicate direct quotes from your manuscript), or elaborate on this item by providing additional information not in the ms, or briefly explain why the item is not applicable/relevant for your study

Patients were randomised electronically as all information is on a database, including intervention.

Mechanism used to implement the random allocation (9 sequence (such as sequentially numbered containers), describing any steps taken to conceal the sequence until interventions were assigned

בחרת לערוך את התשובה שלך. שיתוף כתובת האתר הזו יאפשר גם לאחרים לערוך את התשובה שלך.

הוספת תשובה חדשה

Does your paper address CONSORT subitem 9? *

Copy and paste relevant sections from the manuscript (include quotes in quotation marks "like this" to indicate direct quotes from your manuscript), or elaborate on this item by providing additional information not in the ms, or briefly explain why the item is not applicable/relevant for your study

Patients were randomised electronically as all information is on a database, including intervention.

Who generated the random allocation sequence, who (10 enrolled participants, and who assigned participants to interventions

Does your paper address CONSORT subitem 10? *

Copy and paste relevant sections from the manuscript (include quotes in quotation marks "like this" to indicate direct quotes from your manuscript), or elaborate on this item by providing additional information not in the ms, or briefly explain why the item is not applicable/relevant for your study

Patients were randomised electronically as all information is on a database, including intervention.

11a) If done, who was blinded after assignment to interventions (for example, participants, care providers, those assessing outcomes) and how

NPT: Whether or not administering co-interventions were blinded to group assignment

11a-i) Specify who was blinded, and who wasn't

Specify who was blinded, and who wasn't. Usually, in web-based trials it is not possible to blind the participants [1, 3] (this should be clearly acknowledged), but it may be possible to blind outcome assessors, those doing data analysis or those administering co-interventions (if any).

	5	4	3	2	1	
essential	○	○	○	◉	○	subitem not at all important

Does your paper address subitem 11a-i? *

Copy and paste relevant sections from the manuscript (include quotes in quotation marks "like this" to indicate direct quotes from your manuscript), or elaborate on this item by providing additional information not in the ms, or briefly explain why the item is not applicable/relevant for your study

בחרת לערוך את התשובה שלך. שיתוף כתובת האתר הזו יאפשר גם לאחרים לערוך את התשובה שלך.

הוספת תשובה חדשה

11a-ii) Discuss e.g., whether participants knew which intervention was the “intervention of interest” and which one was the “comparator”

Informed consent procedures (4a-ii) can create biases and certain expectations - discuss e.g., whether participants knew which intervention was the “intervention of interest” and which one was the “comparator”.

	5	4	3	2	1	
essential	○	◉	○	○	○	subitem not at all important

Does your paper address subitem 11a-ii?

Copy and paste relevant sections from the manuscript (include quotes in quotation marks "like this" to indicate direct quotes from your manuscript), or elaborate on this item by providing additional information not in the ms, or briefly explain why the item is not applicable/relevant for your study

"As the SMS messages were sent automatically to a randomly selected population, the patients in the control group were not aware of the intervention. "

11b) If relevant, description of the similarity of interventions

this item is usually not relevant for ehealth trials as it refers to similarity of a placebo or) (sham intervention to a active medication/intervention

Does your paper address CONSORT subitem 11b? *

Copy and paste relevant sections from the manuscript (include quotes in quotation marks "like this" to indicate direct quotes from your manuscript), or elaborate on this item by providing additional information not in the ms, or briefly explain why the item is not applicable/relevant for your study

N/A

12a) Statistical methods used to compare groups for primary and secondary outcomes

NPT: When applicable, details of whether and how the clustering by care providers or centers was addressed

בחרת לערוך את התשובה שלך. שיתוף כתובת האתר הזו יאפשר גם לאחרים לערוך את התשובה שלך.

הוספת תשובה חדשה

Does your paper address CONSORT subitem 12a? *

Copy and paste relevant sections from the manuscript (include quotes in quotation marks "like this" to indicate direct quotes from your manuscript), or elaborate on this item by providing additional information not in the ms, or briefly explain why the item is not applicable/relevant for your study

"We compared demographic variables between the intervention and control groups using chi-squared for discrete variables. Logistic regression was used to calculate odds ratios (ORs) and 95% confidence intervals (CIs) for the rate of colonoscopy within 120 days of receiving a positive FOBT result, adjusting for main potential and variables found to be significantly related to adherence in univariate analysis."

12a-i) Imputation techniques to deal with attrition / missing values

Imputation techniques to deal with attrition / missing values: Not all participants will use the intervention/comparator as intended and attrition is typically high in ehealth trials. Specify how participants who did not use the application or dropped out from the trial were treated in the statistical analysis (a complete case analysis is strongly discouraged, and simple imputation techniques such as LOCF may also be problematic [4]).

	5	4	3	2	1	
essential	○	○	○	○	◉	subitem not at all important

Does your paper address subitem 12a-i? *

Copy and paste relevant sections from the manuscript (include quotes in quotation marks "like this" to indicate direct quotes from your manuscript), or elaborate on this item by providing additional information not in the ms, or briefly explain why the item is not applicable/relevant for your study

As text messages were "pushed" we had no attrition

12b) Methods for additional analyses, such as subgroup analyses and adjusted analyses

בחרת לערוך את התשובה שלך. שיתוף כתובת האתר הזו יאפשר גם לאחרים לערוך את התשובה שלך.

הוספת תשובה חדשה

Does your paper address CONSORT subitem 12b? *

Copy and paste relevant sections from the manuscript (include quotes in quotation marks "like this" to indicate direct quotes from your manuscript), or elaborate on this item by providing additional information not in the ms, or briefly explain why the item is not applicable/relevant for your study

"We compared demographic variables between the intervention and control groups using chi-squared for discrete variables. Logistic regression was used to calculate odds ratios (ORs) and 95% confidence intervals (CIs) for the rate of colonoscopy within 120 days of receiving a positive FOBT result, adjusting for main potential and variables found to be significantly related to adherence in univariate analysis."

X26) REB/IRB Approval and Ethical Considerations [recommended as subheading under "Methods"] (not a (CONSORT item

X26-i) Comment on ethics committee approval

	5	4	3	2	1	
essential	(selected)					subitem not at all important

Does your paper address subitem X26-i?

Copy and paste relevant sections from the manuscript (include quotes in quotation marks "like this" to indicate direct quotes from your manuscript), or elaborate on this item by providing additional information not in the ms, or briefly explain why the item is not applicable/relevant for your study

"The study was approved by the Meuhedet Institutional Review Board (trial reference number: 01-023-03-16)."

x26-ii) Outline informed consent procedures

Outline informed consent procedures e.g., if consent was obtained offline or online (how? Checkbox, etc.?), and what information was provided (see 4a-ii). See [6] for some items to be included in informed consent documents.

	5	4	3	2	1	
essential			(selected)			subitem not at all important

בחרת לערוך את התשובה שלך. שיתוף כתובת האתר הזו יאפשר גם לאחרים לערוך את התשובה שלך.

הוספת תשובה חדשה

Does your paper address subitem X26-ii?

Copy and paste relevant sections from the manuscript (include quotes in quotation marks "like this" to indicate direct quotes from your manuscript), or elaborate on this item by providing additional information not in the ms, or briefly explain why the item is not applicable/relevant for your study

"The study was exempt from informed consent, and only agreement to receive text messages was required. "

X26-iii) Safety and security procedures

Safety and security procedures, incl. privacy considerations, and any steps taken to reduce the likelihood or detection of harm (e.g., education and training, availability of a hotline)

	5	4	3	2	1	
essential	○	○	○	○	◉	subitem not at all important

Does your paper address subitem X26-iii?

Copy and paste relevant sections from the manuscript (include quotes in quotation marks "like this" to indicate direct quotes from your manuscript), or elaborate on this item by providing additional information not in the ms, or briefly explain why the item is not applicable/relevant for your study

N/A

RESULTS

13a) For each group, the numbers of participants who were randomly assigned, received intended treatment, and were analysed for the primary outcome

NPT: The number of care providers or centers performing the intervention in each group and the number of patients treated by each care provider in each center

Does your paper address CONSORT subitem 13a? *

Copy and paste relevant sections from the manuscript (include quotes in quotation marks "like this" to indicate direct quotes from your manuscript), or elaborate on this item by providing additional information not in the ms, or briefly explain why the item is not applicable/relevant for your study

"A total of 470 eligible patients were randomized 232 (49.36%) in the intervention group and 238 (50.64%) in the control group. "

בחרת לערוך את התשובה שלך. שיתוף כתובת האתר הזו יאפשר גם לאחרים לערוך את התשובה שלך.

הוספת תשובה חדשה

13b) For each group, losses and exclusions after randomisation, together with reasons

Does your paper address CONSORT subitem 13b? (NOTE: Preferably, this is shown in a CONSORT flow diagram) *

Copy and paste relevant sections from the manuscript (include quotes in quotation marks "like this" to indicate direct quotes from your manuscript), or elaborate on this item by providing additional information not in the ms, or briefly explain why the item is not applicable/relevant for your study

"Fifty-two (18%) patients were excluded from the control group, 20 because of family history of CRC, 2 died during the study, 16 had an oncology diagnosis and 14 had a colonoscopy prior to the positive FOBT. Eighty-seven (27.2%) patients were excluded from the intervention group, 26 because of family history of CRC, 6 had an oncology diagnosis, 12 had a colonoscopy prior to the positive FOBT, and 43 refused to receive an SMS after the initial approval or they were unable to receive SMS although they agreed to receive it." and Figure 1

13b-i) Attrition diagram

Strongly recommended: An attrition diagram (e.g., proportion of participants still logging in or using the intervention/comparator in each group plotted over time, similar to a survival curve) or other figures or tables demonstrating usage/dose/engagement.

	5	4	3	2	1	
essential	○	○	○	◉	○	subitem not at all important

Does your paper address subitem 13b-i?

Copy and paste relevant sections from the manuscript or cite the figure number if applicable (include quotes in quotation marks "like this" to indicate direct quotes from your manuscript), or elaborate on this item by providing additional information not in the ms, or briefly explain why the item is not applicable/relevant for your study

"43 refused to receive an SMS after the initial approval or they were unable to receive SMS although they agreed to receive it."

14a) Dates defining the periods of recruitment and follow-up

בחרת לערוך את התשובה שלך. שיתוף כתובת האתר הזו יאפשר גם לאחרים לערוך את התשובה שלך.

הוספת תשובה חדשה

Does your paper address CONSORT subitem 14a? *

Copy and paste relevant sections from the manuscript (include quotes in quotation marks "like this" to indicate direct quotes from your manuscript), or elaborate on this item by providing additional information not in the ms, or briefly explain why the item is not applicable/relevant for your study

"In 2016 3,397 patients aged 50-75 had a positive FOBT result. Of these, A total of 609 patients from Meuhedet who had a positive FOBT in 2016 were randomly selected and allocated were randomized to intervention and control groups;"

14a-i) Indicate if critical “secular events” fell into the study period

Indicate if critical “secular events” fell into the study period, e.g., significant changes in Internet resources available or “changes in computer hardware or Internet delivery resources”

	5	4	3	2	1	
essential	○	○	○	○	●	subitem not at all important

Does your paper address subitem 14a-i?

Copy and paste relevant sections from the manuscript (include quotes in quotation marks "like this" to indicate direct quotes from your manuscript), or elaborate on this item by providing additional information not in the ms, or briefly explain why the item is not applicable/relevant for your study

N/A

(14b) Why the trial ended or was stopped (early

Does your paper address CONSORT subitem 14b? *

Copy and paste relevant sections from the manuscript (include quotes in quotation marks "like this" to indicate direct quotes from your manuscript), or elaborate on this item by providing additional information not in the ms, or briefly explain why the item is not applicable/relevant for your study

N/A

A table showing baseline demographic and clinical (15 characteristics for each group

NPT: When applicable, a description of care providers (case volume, qualification, expertise, etc.) and centers (volume) in each group

בחרת לערוך את התשובה שלך. שיתוף כתובת האתר הזו יאפשר גם לאחרים לערוך את התשובה שלך.
הוספת תשובה חדשה

Does your paper address CONSORT subitem 15? *

Copy and paste relevant sections from the manuscript (include quotes in quotation marks "like this" to indicate direct quotes from your manuscript), or elaborate on this item by providing additional information not in the ms, or briefly explain why the item is not applicable/relevant for your study

Table 1

15-i) Report demographics associated with digital divide issues

In ehealth trials it is particularly important to report demographics associated with digital divide issues, such as age, education, gender, social-economic status, computer/Internet/ehealth literacy of the participants, if known.

	5	4	3	2	1	
essential	○	◉	○	○	○	subitem not at all important

Does your paper address subitem 15-i? *

Copy and paste relevant sections from the manuscript (include quotes in quotation marks "like this" to indicate direct quotes from your manuscript), or elaborate on this item by providing additional information not in the ms, or briefly explain why the item is not applicable/relevant for your study

"We also examined potential confounders such as gender, age, SES, ethnicity, and district "

For each group, number of participants (denominator) (16 included in each analysis and whether the analysis was by original assigned groups

16-i) Report multiple “denominators” and provide definitions

Report multiple “denominators” and provide definitions: Report N’s (and effect sizes) “across a range of study participation [and use] thresholds” [1], e.g., N exposed, N consented, N used more than x times, N used more than y weeks, N participants “used” the intervention/comparator at specific pre-defined time points of interest (in absolute and relative numbers per group). Always clearly define “use” of the intervention.

	5	4	3	2	1	
essential	◉	○	○	○	○	subitem not at all important

בחרת לערוך את התשובה שלך. שיתוף כתובת האתר הזו יאפשר גם לאחרים לערוך את התשובה שלך.

הוספת תשובה חדשה

Does your paper address subitem 16-i? *

Copy and paste relevant sections from the manuscript (include quotes in quotation marks "like this" to indicate direct quotes from your manuscript), or elaborate on this item by providing additional information not in the ms, or briefly explain why the item is not applicable/relevant for your study

"Of 232 patients in the intervention group (who received a text reminder), 163 had a colonoscopy within 120 days of a positive fecal occult blood test, and 112 of 238 had one in the control group (70% vs. 47%, P<.0001). "

16-ii) Primary analysis should be intent-to-treat

Primary analysis should be intent-to-treat, secondary analyses could include comparing only "users", with the appropriate caveats that this is no longer a randomized sample (see 18-i).

	5	4	3	2	1	
essential	○	○	○	◉	○	subitem not at all important

Does your paper address subitem 16-ii?

Copy and paste relevant sections from the manuscript (include quotes in quotation marks "like this" to indicate direct quotes from your manuscript), or elaborate on this item by providing additional information not in the ms, or briefly explain why the item is not applicable/relevant for your study

"We conducted the analysis including the 43 patients who did not receive the SMS, and found that adherence rates remained significantly higher both in the univariate (63.5% vs 47.1 in control group), and in the multivariable model (OR 2.04, 95%CI 1.387-2.993, p<0.0001)."

17a) For each primary and secondary outcome, results for each group, and the estimated effect size and its precision ((such as 95% confidence interval

Does your paper address CONSORT subitem 17a? *

Copy and paste relevant sections from the manuscript (include quotes in quotation marks "like this" to indicate direct quotes from your manuscript), or elaborate on this item by providing additional information not in the ms, or briefly explain why the item is not applicable/relevant for your study

"Variable :Intervention vs. Control, Odds ratio 2.9, 95% Confidence interval 1.92- 4.48, p-value .001" (from Table 3)

בחרת לערוך את התשובה שלך. שיתוף כתובת האתר הזו יאפשר גם לאחרים לערוך את התשובה שלך.

הוספת תשובה חדשה

17a-i) Presentation of process outcomes such as metrics of use and intensity of use

In addition to primary/secondary (clinical) outcomes, the presentation of process outcomes such as metrics of use and intensity of use (dose, exposure) and their operational definitions is critical. This does not only refer to metrics of attrition (13-b) (often a binary variable), but also to more continuous exposure metrics such as "average session length". These must be accompanied by a technical description how a metric like a "session" is defined (e.g., timeout after idle time) [1] (report under item 6a).

	5	4	3	2	1	
essential	○	○	◉	○	○	subitem not at all important

Does your paper address subitem 17a-i?

Copy and paste relevant sections from the manuscript (include quotes in quotation marks "like this" to indicate direct quotes from your manuscript), or elaborate on this item by providing additional information not in the ms, or briefly explain why the item is not applicable/relevant for your study

The intervention was not user-dependent: "Each patient in the intervention group also received an automated text message up to a week after the positive FOBT result. The text read: "Hello. There is a lab test result ready for you. Contact your physician for an explanation of the findings." Two additional automated text message reminders were sent to the patient at 2 weeks and 1 month reading, "Hello, This is a reminder. It is essential that you contact your physician if you have not already done so."."

17b) For binary outcomes, presentation of both absolute and relative effect sizes is recommended

Does your paper address CONSORT subitem 17b? *

Copy and paste relevant sections from the manuscript (include quotes in quotation marks "like this" to indicate direct quotes from your manuscript), or elaborate on this item by providing additional information not in the ms, or briefly explain why the item is not applicable/relevant for your study

"Of 232 patients in the intervention group (who received a text reminder), 163 had a colonoscopy within 120 days of a positive fecal occult blood test, and 112 of 238 had one in the control group (70% vs. 47%, P<.0001). "

Results of any other analyses performed, including (18 subgroup analyses and adjusted analyses, distinguishing pre-

בחרת לערוך את התשובה שלך. שיתוף כתובת האתר הזו יאפשר גם לאחרים לערוך את התשובה שלך.

הוספת תשובה חדשה

Does your paper address CONSORT subitem 18? *

Copy and paste relevant sections from the manuscript (include quotes in quotation marks "like this" to indicate direct quotes from your manuscript), or elaborate on this item by providing additional information not in the ms, or briefly explain why the item is not applicable/relevant for your study

"Of 232 patients in the intervention group (who received a text reminder), 163 had a colonoscopy within 120 days of a positive fecal occult blood test, and 112 of 238 had one in the control group (70% vs. 47%, P<.0001). " and "The adjusted likelihood of adherence to colonoscopy was 2.5 higher in the intervention group after adjusting for confounding factorsOR for the intervention group vs. the control group was 2.9 (95%CI: 1.92-4.48; p=0.001)."

18-i) Subgroup analysis of comparing only users

A subgroup analysis of comparing only users is not uncommon in ehealth trials, but if done, it must be stressed that this is a self-selected sample and no longer an unbiased sample from a randomized trial (see 16-iii).

	5	4	3	2	1	
essential	○	○	○	○	◉	subitem not at all important

Does your paper address subitem 18-i?

Copy and paste relevant sections from the manuscript (include quotes in quotation marks "like this" to indicate direct quotes from your manuscript), or elaborate on this item by providing additional information not in the ms, or briefly explain why the item is not applicable/relevant for your study

As the only difference (Table 2) beween the intervention and control groups was the intervention and the outcome, we did not present this analysis

All important harms or unintended effects in each group (19

(for specific guidance see CONSORT for harms)

Does your paper address CONSORT subitem 19? *

Copy and paste relevant sections from the manuscript (include quotes in quotation marks "like this" to indicate direct quotes from your manuscript), or elaborate on this item by providing additional information not in the ms, or briefly explain why the item is not applicable/relevant for your study

The intervention is sending three text messages to paatients who agreed to receive text messages to their personal mobile phone. We did not perceive there to be a risk. This was supported by the IRB.

בחרת לערוך את התשובה שלך. שיתוף כתובת האתר הזו יאפשר גם לאחרים לערוך את התשובה שלך.

הוספת תשובה חדשה

19-i) Include privacy breaches, technical problems

Include privacy breaches, technical problems. This does not only include physical “harm” to participants, but also incidents such as perceived or real privacy breaches [1], technical problems, and other unexpected/unintended incidents. “Unintended effects” also includes unintended positive effects [2].

	5	4	3	2	1	
essential	◉	○	○	○	○	subitem not at all important

Does your paper address subitem 19-i?

Copy and paste relevant sections from the manuscript (include quotes in quotation marks "like this" to indicate direct quotes from your manuscript), or elaborate on this item by providing additional information not in the ms, or briefly explain why the item is not applicable/relevant for your study

As a healthcare organization privacy is of extreme importance. All our database activities are monitored continouosly for breaches of privacy.

19-ii) Include qualitative feedback from participants or observations from staff/researchers

Include qualitative feedback from participants or observations from staff/researchers, if available, on strengths and shortcomings of the application, especially if they point to unintended/unexpected effects or uses. This includes (if available) reasons for why people did or did not use the application as intended by the developers.

	5	4	3	2	1	
essential	○	○	○	○	◉	subitem not at all important

Does your paper address subitem 19-ii?

Copy and paste relevant sections from the manuscript (include quotes in quotation marks "like this" to indicate direct quotes from your manuscript), or elaborate on this item by providing additional information not in the ms, or briefly explain why the item is not applicable/relevant for your study

N/A

DISCUSSION

Interpretation consistent with results, balancing benefits (22 and harms, and considering other relevant evidence

בחרת לערוך את התשובה שלך. שיתוף כתובת האתר הזו יאפשר גם לאחרים לערוך את התשובה שלך.

הוספת תשובה חדשה

22-i) Restate study questions and summarize the answers suggested by the data, starting with primary outcomes and process outcomes (use)

Restate study questions and summarize the answers suggested by the data, starting with primary outcomes and process outcomes (use).

	5	4	3	2	1	
essential	(●)	( )	( )	( )	( )	subitem not at all important

Does your paper address subitem 22-i? *

Copy and paste relevant sections from the manuscript (include quotes in quotation marks "like this" to indicate direct quotes from your manuscript), or elaborate on this item by providing additional information not in the ms, or briefly explain why the item is not applicable/relevant for your study

"we have shown that sending SMS reminders to patients following a positive FOBT is an effective way to increase adherence rates to follow-up colonoscopy."

22-ii) Highlight unanswered new questions, suggest future research

Highlight unanswered new questions, suggest future research.

	5	4	3	2	1	
essential	(●)	( )	( )	( )	( )	subitem not at all important

Does your paper address subitem 22-ii?

Copy and paste relevant sections from the manuscript (include quotes in quotation marks "like this" to indicate direct quotes from your manuscript), or elaborate on this item by providing additional information not in the ms, or briefly explain why the item is not applicable/relevant for your study

"We have shown that a simple, inexpensive intervention for patients improves colonoscopy follow-up after a positive FOBT. It is important to maximize the potential of these findings by increasing the acceptance of text messages within the population and to examine their use in other screening programs. It is also important to examine in future studies the reasons that patients refuse to receive text messages. "

Trial limitations, addressing sources of potential bias (20 [illegible]

הוספת תשובה חדשה

בחרת לערוך את התשובה שלך. שיתוף כתובת האתר הזו יאפשר גם לאחרים לערוך את התשובה שלך.

20-i) Typical limitations in ehealth trials

Typical limitations in ehealth trials: Participants in ehealth trials are rarely blinded. Ehealth trials often look at a multiplicity of outcomes, increasing risk for a Type I error. Discuss biases due to non-use of the intervention/usability issues, biases through informed consent procedures, unexpected events.

	5	4	3	2	1	
essential	◉	○	○	○	○	subitem not at all important

Does your paper address subitem 20-i? *

Copy and paste relevant sections from the manuscript (include quotes in quotation marks "like this" to indicate direct quotes from your manuscript), or elaborate on this item by providing additional information not in the ms, or briefly explain why the item is not applicable/relevant for your study

"A limitation of this study is that only patients who agreed to receive text messages were included in the study, which created a biased population in terms of age, cultural beliefs, and SES. "

Generalisability (external validity, applicability) of the trial (21 findings

NPT: External validity of the trial findings according to the intervention, comparators, patients, and care providers or centers involved in the trial

21-i) Generalizability to other populations

Generalizability to other populations: In particular, discuss generalizability to a general Internet population, outside of a RCT setting, and general patient population, including applicability of the study results for other organizations

	5	4	3	2	1	
essential	○	◉	○	○	○	subitem not at all important

בחרת לערוך את התשובה שלך. שיתוף כתובת האתר הזו יאפשר גם לאחרים לערוך את התשובה שלך.

הוספת תשובה חדשה

Does your paper address subitem 21-i?

Copy and paste relevant sections from the manuscript (include quotes in quotation marks "like this" to indicate direct quotes from your manuscript), or elaborate on this item by providing additional information not in the ms, or briefly explain why the item is not applicable/relevant for your study

"This study was conducted in one of four healthcare provider organisations in Israel. Selection of providers is voluntary, and our member distribution in terms of age, gender and SES are similar to the Israeli population. In addition, the Israeli population is very ethnically diverse, and includes immigrants from many countries. Our findings are therefore generalisable to other populations."

21-ii) Discuss if there were elements in the RCT that would be different in a routine application setting

Discuss if there were elements in the RCT that would be different in a routine application setting (e.g., prompts/reminders, more human involvement, training sessions or other co-interventions) and what impact the omission of these elements could have on use, adoption, or outcomes if the intervention is applied outside of a RCT setting.

	5	4	3	2	1	
essential	○	○	○	○	◉	subitem not at all important

Does your paper address subitem 21-ii?

Copy and paste relevant sections from the manuscript (include quotes in quotation marks "like this" to indicate direct quotes from your manuscript), or elaborate on this item by providing additional information not in the ms, or briefly explain why the item is not applicable/relevant for your study

Our intervention is feasible in routine applications, inexpensive, and does not depend on any other factors.

OTHER INFORMATION

Registration number and name of trial registry (23

Does your paper address CONSORT subitem 23? *

Copy and paste relevant sections from the manuscript (include quotes in quotation marks "like this" to indicate direct quotes from your manuscript), or elaborate on this item by providing additional information not in the ms, or briefly explain why the item is not applicable/relevant for your study

NCT03642652

בחרת לערוך את התשובה שלך. שיתוף כתובת האתר הזו יאפשר גם לאחרים לערוך את התשובה שלך.

הוספת תשובה חדשה

Where the full trial protocol can be accessed, if available (24

Does your paper address CONSORT subitem 24? *

Cite a Multimedia Appendix, other reference, or copy and paste relevant sections from the manuscript (include quotes in quotation marks "like this" to indicate direct quotes from your manuscript), or elaborate on this item by providing additional information not in the ms, or briefly explain why the item is not applicable/relevant for your study

https://clinicaltrials.gov/ct2/home

Sources of funding and other support (such as supply of (25 drugs), role of funders

Does your paper address CONSORT subitem 25? *

Copy and paste relevant sections from the manuscript (include quotes in quotation marks "like this" to indicate direct quotes from your manuscript), or elaborate on this item by providing additional information not in the ms, or briefly explain why the item is not applicable/relevant for your study

No funding or support was received.

(X27) Conflicts of Interest (not a CONSORT item

X27-i) State the relation of the study team towards the system being evaluated

In addition to the usual declaration of interests (financial or otherwise), also state the relation of the study team towards the system being evaluated, i.e., state if the authors/evaluators are distinct from or identical with the developers/sponsors of the intervention.

	5	4	3	2	1	
essential	○	○	○	○	◉	subitem not at all important

Does your paper address subitem X27-i?

Copy and paste relevant sections from the manuscript (include quotes in quotation marks "like this" to indicate direct quotes from your manuscript), or elaborate on this item by providing additional information not in the ms, or briefly explain why the item is not applicable/relevant for your study

This study was part of our role in the organisation, and aimed at improving adherence. part of the organizational agenda.

בחרת לערוך את התשובה שלך. שיתוף כתובת האתר הזו יאפשר גם לאחרים לערוך את התשובה שלך.

הוספת תשובה חדשה

About the CONSORT EHEALTH checklist

As a result of using this checklist, did you make changes in your manuscript? *

- ○ yes, major changes
- ◉ yes, minor changes
- ○ no

What were the most important changes you made as a result of using this checklist?

Added a recommendation for further study.

How much time did you spend on going through the checklist INCLUDING making changes in your manuscript *

5 hours

As a result of using this checklist, do you think your manuscript has improved? *

- ◉ yes
- ○ no
- ○ אחר:

Would you like to become involved in the CONSORT EHEALTH group?

This would involve for example becoming involved in participating in a workshop and writing an "Explanation and Elaboration" document

- ○ yes
- ◉ no

בחרת לערוך את התשובה שלך. שיתוף כתובת האתר הזו יאפשר גם לאחרים לערוך את התשובה שלך.

הוספת תשובה חדשה

Any other comments or questions on CONSORT EHEALTH

התשובה שלך

STOP - Save this form as PDF before you click submit

To generate a record that you filled in this form, we recommend to generate a PDF of this .page (on a Mac, simply select "print" and then select "print as PDF") before you submit it

.When you submit your (revised) paper to JMIR, please upload the PDF as supplementary file

Don't worry if some text in the textboxes is cut off, as we still have the complete information !in our database. Thank you

! Final step: Click submit

!Click submit so we have your answers in our database

שלח

לעולם אל תשלח סיסמאות באמצעות Google Forms.

תוכן זה לא נוצר ולא נתמך על ידי Google. דיווח על שימוש לרעה - תנאים והגבלות - מונחים נוספים

Google Forms

בחרת לערוך את התשובה שלך. שיתוף כתובת האתר הזו יאפשר גם לאחרים לערוך את התשובה שלך.

הוספת תשובה חדשה
